# Differential *In Vivo* Tumorigenicity of Distinct Subpopulations from a Luminal-Like Breast Cancer Xenograft

**DOI:** 10.1371/journal.pone.0113278

**Published:** 2014-11-24

**Authors:** Nirma Skrbo, Geir-Olav Hjortland, Alexandr Kristian, Ruth Holm, Silje Nord, Lina Prasmickaite, Olav Engebraaten, Gunhild M. Mælandsmo, Therese Sørlie, Kristin Andersen

**Affiliations:** 1 Department of Tumor Biology, Institute for Cancer Research, The Norwegian Radium Hospital, Oslo University Hospital, Oslo, Norway; 2 Department of Genetics, Institute for Cancer Research, The Norwegian Radium Hospital, Oslo University Hospital, Oslo, Norway; 3 Department of Pathology, The Norwegian Radium Hospital, Oslo University Hospital, Oslo, Norway; 4 Cancer Stem Cell Innovation Centre, Oslo University Hospital, The Norwegian Radium Hospital, Oslo, Norway; 5 Department of Pharmacy, Faculty of Health Sciences, University of Tromsø, Tromsø, Norway; 6 Department of Oncology, Oslo University Hospital, Oslo, Norway; 7 Institute for Clinical Medicine, Faculty of Medicine, University of Oslo, Oslo, Norway; 8 K.G. Jebsen Centre for Breast Cancer Research, Institute for Clinical Medicine, University of Oslo, Oslo, Norway; University of Alabama at Birmingham, United States of America

## Abstract

Intratumor heterogeneity caused by genetic, phenotypic or functional differences between cancer cell subpopulations is a considerable clinical challenge. Understanding subpopulation dynamics is therefore central for both optimization of existing therapy and for development of new treatment. The aim of this study was to isolate subpopulations from a primary tumor and by comparing molecular characteristics of these subpopulations, find explanations to their differing tumorigenicity. Cell subpopulations from two patient derived *in vivo* models of primary breast cancer, ER+ and ER-, were identified. EpCAM+ cells from the ER+ model gave rise to tumors independently of stroma cell support. The tumorigenic fraction was further divided based on SSEA-4 and CD24 expression. Both markers were expressed in ER+ breast cancer biopsies. FAC-sorted cells based on EpCAM, SSEA-4 and CD24 expression were subsequently tested for differences in functionality by *in vivo* tumorigenicity assay. Three out of four subpopulations of cells were tumorigenic and showed variable ability to recapitulate the marker expression of the original tumor. Whole genome expression analysis of the sorted populations disclosed high similarity in the transcriptional profiles between the tumorigenic populations. Comparing the non-tumorigenic vs the tumorigenic populations, 44 transcripts were, however, significantly differentially expressed. A subset of these, 26 identified and named genes, highly expressed in the non-tumorigenic population, predicted longer overall survival (N = 737, p<0.0001) and distant metastasis free survival (DMFS) (N = 1379, p<0.0001) when performing Kaplan-Meier survival analysis using the GOBO online database. The 26 gene set correlated with longer DMFS in multiple breast cancer subgroups. Copy number profiling revealed no aberrations that could explain the observed differences in tumorigenicity. This study emphasizes the functional variability among cell populations that are otherwise genomically similar, and that the risk of breast cancer recurrence can only be eliminated if the tumorigenic abilities in multiple cancer cell subpopulations are inhibited.

## Introduction

Cancer cells evading the administered treatment represent the major challenge in oncology. To understand how some cancer cells are able to escape and cause recurrence, researchers have compared primary tumors to small ecosystems where the extracellular components determine the physical environment, and all cell populations, both normal and neoplastic, represent the diversity of the species within the system [1],[2]. Large intratumoral cellular diversity ensures that at least one tumor cell subpopulation is able to tolerate the altered conditions, during treatment, or relocation to a metastatic site [2],[3]. Our challenge is to understand why heterogeneity is sustained in the developing tumor, and how to best eradicate dynamically changing cancer cell populations before they develop strategies to withstand attacks from various treatment. Details of cancer cell population dynamics is obviously not possible to study in tissue derived directly from patients. The best option is therefore, clinically representative patient derived xenograft models (PDX), that has not been subjected to *in vitro* culture differentiation or selection [4]. Such models provide stable access to primary tumor material enabling repeated experiments on the same primary tumor, and thus broad characterization of tumor cell subpopulations. PDX stably recapitulate the molecular composition and the heterogeneity of the mother tumor [4],[5],[6]. The luminal-like PDX used in this study is unique in that it recapitulates estrogen dependency for growth [7],[8].

Although intratumor heterogeneity is well established, its origin has been heavily debated. The clonal evolution model was counteracted by the theory of “cancer stem cells” or tumor initiating cells, TICs. Several studies in cell lines [9] and animal models [10],[11] have indeed indicated the existence of tumor cell subpopulations with enhanced tumorigenic capacity, compared to the “bulk” tumor cells. Regardless of the origin of TIC populations, identification and functional characterization of both TICs and the seemingly less tumorigenic subpopulations are essential for development of more efficient anti-cancer therapies. It is important to consider that within the ecosystem of a tumor, the TICs and the apparently less tumorigenic cancer cell populations might in fact be equally dependent on each other [12]. Less tumorigenic populations might constitute a reservoir for development of treatment resistance. Clarification of the reciprocal relationships between cell populations within breast tumors, and the dynamics of their differentiation, is therefore needed. The aim of this study was to find phenotypically different subpopulations within a primary tumor that could initiate tumor growth independently of each other, and by comparing molecular characteristics of these subpopulations, find explanations to their diverging capacity. Another objective was to acquire detailed knowledge on functional differences, cell surface marker expression, and molecular portraits of tumorigenic subpopulations within a primary breast cancer model, to open the possibility for future, controlled studies of cancer cell population dynamics and cellular plasticity in response to changing conditions. In the present manuscript, subpopulations from two uniquely well characterized PDX models [7],[8],[13],[14],[15],[16] were therefore defined, and their cellular surface marker composition was elucidated. To prospectively characterize the intratumoral heterogeneity; flow cytometry was combined with *in vivo* tumorigenicity assay and immunohistochemistry (IHC). In addition, four subpopulations from the luminal like ER+ PDX model were subjected to molecular comparisons using whole genome expression profiling and SNP array analysis of genomic aberrations.

## Materials and Methods

### Ethic statement

The MAS98.12 and MAS98.06 tumor models were established by implantation of biopsy tissues from primary mammary carcinomas as previously described (Approved by the Norwegian Regional Committee for Medical Research Ethics, Health region II (reference number 2.2007.2155) [7],[16]. Informed written consent was obtained from all patients.

All procedures and experiments involving animals were approved by the National Animal Research Authority (http://www.fdu.no/), and were conducted according to the regulations of the Federation of European Laboratory Animal Science Association (FELASA). All surgery was performed under sevofluran anaesthesia, and all efforts were made to minimize suffering.

### Patient derived breast cancer xenografts models (PDX)

Both the primary carcinomas and the xenograft models have been characterized using gene expression profiling. These analyses demonstrated that the primary carcinomas could be classified as luminal-like and basal-like subtypes of breast cancer, and that these molecular subtypes were retained in the MAS98.06 (ER+, luminal-like) and MAS98.12 (ER-, basal-like) xenografts. Relevant characteristics of the models are presented in [16]. Both models are now routinely serially transplanted without enzymatic digestion, as 1-3mm^3^ pieces in nude (athymic) mice. Notably, to optimize the conditions for tumorigenicity [17] the *in vivo* tumorigenicity assays using dissociated tumor cells in suspension were performed using NOD/SCID interleukin-2 receptor gamma chain null (Il2rg-/-) (NSG) mice. Mice were kept under pathogen-free conditions, at constant temperature (21.5±0.5°C) and humidity (55±5%), 20 air changes/hr and a 12 hr light/dark cycle. Distilled tap water was given *ad libitum*, supplemented with 17-β-estradiol at a concentration of 4 mg/l. All mice used in the experiment were locally bred at the animal facility of our institute.

### Collection of primary tumor material

The five primary breast cancer samples analysed in this study were collected as part of a study where patients were referred for surgical treatment of breast cancer to several different hospitals in the Oslo region. The study was approved by the Norwegian Regional Committee for Medical Research Ethics, Health region II (reference number S-07278a). All patients have given written consent for the use of material for research purposes.

### Dissociation of tumors to single cell suspension for FACS

Tumors, routinely implanted as 1–2 mm^3^ pieces, bilaterally in mammary fat pads of female athymic nude mice (Athymic Nude-Foxn1^nu^; weight, 23–25 g; age, 12–13 weeks), were harvested when largest tumor diameter reached 10 mm. Tumors were then manually minced and incubated in Collagenase III solution (Collagenase III, Worthington, Lakewood Township, USA (900U/ml), dissolved in DMEM/F12 with 0,5% human serum albumin, 2% Hepes, Pencillin and Streptamycin), for 3 hours at 37°C on an orbital shaker. The digested tumor cells were washed twice in DMEM/F12 medium following a quick spin after the second wash, to precipitate and separate the organoid fraction from the single cell suspension. Following an additional spinning of the single cell suspension, the single cells were either frozen in 90% FCS with 10% DMSO or directly re-suspended in flow blocking buffer for subsequent staining with antibodies and further flow analysis or FACS.

### Fluorescence-activated cell sorting and Flow analysis

Single cell suspensions were diluted in cold staining buffer (PBS containing 0.5% FCS and 3% human immune globulin (Gammaguard) (N.V Baxter S.A, Belgium)) and stained with fluorescently-labelled antibodies, diluted according to the manufacturer's recommendation. Following 30 min incubation at 4°C, the stained cells were washed once with PBS, then re-suspended in PBS with 2% FCS and further analysed by LSRII flow cytometer (Becton Dickinson) using BD FACSDiva™ software. The antibody- stained single cell suspensions, sorted for further re-injection or DNA/RNA extraction were, after the washing step, re-suspended in DMEM/F12 with 0.5% human serum albumin, 2% Hepes, Penicillin and Streptomycin. The cell populations were sorted by FACS DIVA flow cytometer (Becton Dickinson), equipped with a 488nm Argon laser (Coherent) and 633nm HeNe laser (Spectra Physics), distributing cells from each population into a separate tube containing DMEM/F12 with 0.5% human serum albumin, 2% Hepes, Penicillin and Streptomycin. The single cell suspensions were always stained with 1 µg/ml propidium iodide (PI) (Sigma) prior to flow analysis or sorting to exclude the dead cells from the analysis. Fluorescently-conjugated IgG isotype controls (BD Biosciences, Franklin Lakes, USA) and/or unstained controls were used to set the gates. A minimum of 10,000 events from the viable cell population were recorded for each sample. FlowJo 7.6 software was used to analyse the data. Sorted populations were defined as indicated on figures.

### Antibodies

The following antibodies were used for FACS and flow analysis of human tumor cells; anti-CD24-FITC (clone ML5), anti-CD15 (also known as SSEA-1clone W6D3), anti-CD45-FITC (clone HI30), anti-EpCAM-APC (clone 9C4), were all from Biolegend, San Diego, US. Anti-SSEA-4-PE (clone MC813-70), anti-H2kD-FITC (clone SF1-1.1), anti-CD325-PE (also known as N-cadherin clone 8C11), anti-CD44-PerCP-Cy5.5 (clone G-44-26, also known as G26), anti-CD29 (integrin β1, clone 18/CD29), anti-CD184-PE (clone 12G5), anti-CD49B-PE (clone 12F1), anti-CD31-PE (clone WM59), anti-CD166-PE (clone 3A6), anti CD271-PE (also known as p75, clone C40-1457), anti-anti-NG2-FITC (also known as CSPG4, clone 9.2.27), anti-CD90-PE (clone 5E10), anti-CD-34-PE (clone 581), anti-CD117-APC (also known as c-Kit, clone YB5.B8), anti-CD142-PE (clone HTF1) and Annexin V-PE, were all from BD Biosciences, New Jersey, US. Anti-CDV66-PE (clone SF10) and anti-Tra-1-85-PE were both from R&D Systems, Oxon, UK. Anti-CD133/2-PE (clone 293-C3) was from Milteny Biotech, Lund, Sweden.

For immunohistochemistry the following antibodies were used; anti-S100A4 (clone 20.1) [18], anti-ALDH1A1, rabbit polyclonal (cat no ab51028), Abcam, Cambridge, UK, anti-CK19 (clone A-53-B/A2), Abcam, anti-CK-14 (clone LL002), Novacostra Labs. Ltd., Newcastle Upon Tyne, UK, anti-CD49f (clone GoH3), BD Biosciences, anti-Ki-67 (clone Ki-67), anti CD44 (clone DF1485) and anti-EpCAM (clone Moc-31), DAKO, Copenhagen, Denmark,

### Immunohistochemistry

Immunohistochemistry was performed using the Dako EnVision™ + System, Peroxidase (DAB) (K4011, Dako, Glostrup, DenmarK) and Dakoautostainer, or VECTOR M.O.M.™ immunodetection Kit (PK-2200, Vector Laboratories). Sections were deparaffinized and epitopes unmasked using PT-Link (Dako) and EnVision Flex target retrieval solution, high pH or low pH, and then treated with 0.03% hydrogen peroxide (H_2_O_2_) for 5 minutes to block endogenous peroxidase.

After incubation with rabbit polyclonal antibody for 30 minutes at room temperature, the sections were incubated with peroxidase labelled polymer conjugated to goat anti-rabbit for 30 minutes. Tissue was stained for 10 minutes with 3′3-diaminobenzidine tetrahydrochloride (DAB) and then counterstained with haematoxylin, dehydrated, and mounted in Diatex. Negative controls included substitution of the polyclonal primary antibody with antibody diluent.

The specimens were given a sequential incubation with mouse Ig blocking reagent (60 minutes) and working solution of diluent (5 minutes). Excess working solution of diluent was blotted from the slides before incubation with mouse monoclonal antibodies for 30 minutes. The sections were then incubated with biotinylated anti-mouse IgG for 10 minutes and ABC reagents for 5 minutes. Tissue was stained for 10 minutes with DAB and then counterstained with haematoxylin, dehydrated, and mounted in Diatex. Negative controls included substitution of the monoclonal antibody with mouse myeloma protein of the same subclass and concentration as the monoclonal antibody.

### In vivo tumorigenicity assay

EpCAM positive cells isolated by FACS were pelleted by centrifugation, resuspended in 100 µl of PBS and re-counted. Non-viable cells were detected with tryphan blue staining and excluded from the calculations. To test the stringency of the trypan blue staining, aliquots of the subpopulations were subjected to live (calcein AM) and dead dye (propidium iodide), staining and inspected for differences in cell viability using a fluorescent microscope. In addition, Annexin V flow cytometry was performed on the EpCAM positive population. Only the outer perimeter of each population was isolated by FACS, ensuring maximum difference in marker expression levels. The purity of the subpopulations was tested by reanalyzing the sorted fractions by flow cytometry. Each fraction, dissolved in 100 µl PBS, containing 40.000 cells of 98% purity, was injected in the right mammary fat pad of NSG mice. Tumor diameter was measured twice a week, and the experiment was terminated when the diameter reached 12 mm.

### Isolation of RNA and cDNA synthesis for qPCR

All RNA extractions were performed using Trizol Reagent manufacturer's instructions (Invitrogen Life Science, Carlsbad, USA). RNA concentration was routinely assessed on the NanoDrop 1000 instrument (Thermo Fisher Scientific, Waltham, USA). Generally 0.08–1 µg of RNA was reverse transcribed using the qScript cDNA synthesis kit (Quanta BioSciences Inc.) in a volume of 20 µl and then diluted (in dH2O) to 5–10 µg/μl.

### Gene expression analysis

Four subpopulations from the luminal MAS9806 xenograft were sorted by FACS based on the presence of EpCAM, SSEA-4 and CD24 cell surface markers on the epithelial MAS9806 cells. Total RNA was isolated from four biological replicates of each population (totally 16 samples). 50–100 ng of total RNA was amplified and labeled with cy3-CTP following Agilent Low Input Quick Amplification Labeling Kit protocol for One-Color Microarray-Based Gene Expression Analysis. Hybridization was performed according to the manufacturer's protocol (Agilent One-Color Microarray-Based Gene Expression Analysis v6.0) using 1650 ng cy3-labeled cRNA per sample and hybridized onto Whole Human Genome Oligo Microarrays (4x44K, G4112F).

The microarrays were scanned using Agilent Technologies Microarray Scanner (G2505C). Data were extracted from the scanned images using Feature Extraction Software (Agilent Technologies), version 10.7 and protocol GE1-107-Sep09 for mRNA, using default settings and FULL text output. One sample from the double positive cell fraction was removed from the further analysis due to poor data quality. Raw data were uploaded to Gene expression omnibus (GEO) accession number GSE48384.

### Data analysis and statistics

Microarray expression data were filtered for spot quality and quantile normalized using GeneSpring GX Software (Agilent). A final dataset was generated by additionally averaging the signal intensity of multiple unique probes for each gene. This set included data for 24210 unique genes on 15 microarrays from the four cell subpopulations. Qlucore Omics Explorer 2.3 software was used to compare gene expression profiles between different cell subpopulations. A student t-test was performed to identify genes significantly differentially expressed between the non-tumorigenic dbl.high subpopulation and the three tumorigenic subpopulations. Only genes that contributed the most to the variation across the dataset were included in the analysis. Filtering genes by variance (v = 0.2 for t-test) excluded genes having variance lower than v compared to the gene having the largest variance, from the analysis. The calculated *P*-values were adjusted for multiple testing by applying a False Discovery Rate adjustment (FDR = 0.2). Gene expression-based Outcome for Breast cancer Online (GOBO) tool was used for prognostic validation of sets of genes in a pooled breast cancer data set comprising 1881-samples [19]. Association with outcome for the 26 gene set was investigated by Kaplan-Meier analysis using overall survival and distant metastasis-free survival as endpoint and 10-years censoring.

### Validation of microarray data by RT-qPCR:

Real-time quantitative PCR reactions were performed on the iCycler instrument from BioRad (Hercules, CA). All reactions were run in parallel, and all samples were in 25 µl volume. Each primer mix contained 200 nM FAM-labeled probe, 300 nM of each primer and 1× Perfecta qPCR Supermix (Quanta BioSciences Inc, Gaithersburg, MD). Expression of YARS, a t-RNA synthetase, was used for sample validation and normalization of expression. The reference genes (YARS and TBP) had been tested in a panel of cell lines and found to have equal expression per ng of cDNA. All primers have been validated using appropriate controls, and negative and positive controls for all targets were always included in the PCR runs. Primers were designed using the probe finder software available online at the Universal ProbeLibrary assay design Center (http://www.roche-applied-science.com/sis/rtpcr/upl/index.jsp), and all probes are from the UniversalProbe Library collection (both Roche Applied Science).

### Analysis of genomic copy number alterations

To compare genome wide copy number aberration patterns and degree of heterogeneity between and within the four subpopulations, the luminal MAS9806 xenograft was sorted by FACS based on the presence of EpCAM, SSEA4 and CD24 cell surface markers on the epithelial cells. Genomic DNA was isolated using Quiagen kit according to the manufacturer's protocol, and subjected to Illumina HumanOmniExpressExome v1.2 BeadChips (Performed by Aros Applied Biotechnology A/S, Denmark). aData were analysed using GenomeStudio 2011.1 Software, and by R version 3.0.2 (2013-09-25), using the “DNAcopy package” for segmentation. The standard Illumina reference file (HumanOmniExpressExome-8v1-2_A.egt) was used to identify aberrations in relation to a normal genome. To search for aberrations specific for each subpopulation, the four samples were also compared pairvise. For each possible pairing of the four samples (6), a new segmentation was applied in order to more easily see how much the samples differed from each other (data not shown). Raw data were uploaded to Gene Expression Omnibus (GEO) accession number GSE56103.

## Results

### EpCAM expression was a specific and sensitive marker for the human cell population in both PDX models

**Figure 1:**
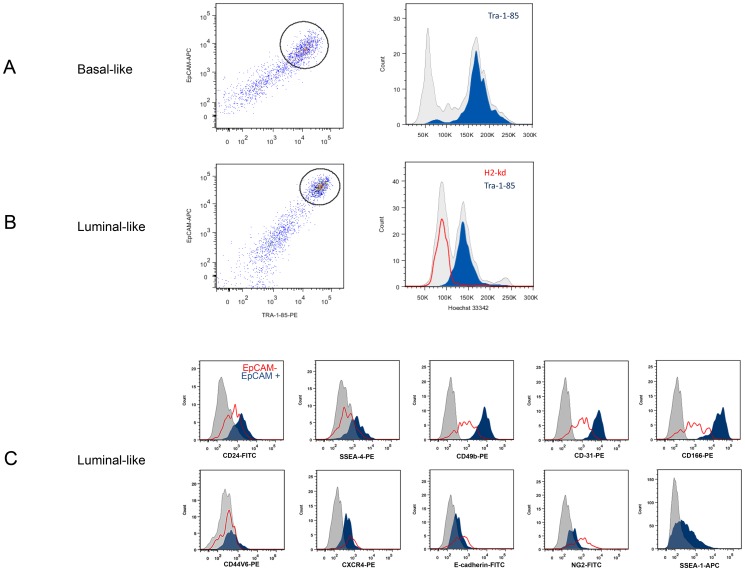
Flow cytometry analysis of total cell fractions of dissociated cells from PDX models. A) Basal-like xenograft cells. B) Luminal-like xenograft cells. A and B displays pseudo-color dot plots (left panels) and histograms (right panels). Freshly harvested xenografts were minced and the whole cell suspensions were washed and stained with monoclonal antibody towards EpCAM, TRA-1-85 (filled blue in histograms), H2-kd (red line in lower histogram) and Hoecst-33342 (intensity measure for DNA content of cells, grey contours in both histograms. Left peak indicate mouse cells, right peak indicate human cells). The population positive for both EpCAM and TRA-1-85, i.e the human tumor cells, are indicated with a circle in the dot plots. C) Flow cytometry analysis of double stained samples (marker of interest and EpCAM/Tra-1-85) of the Luminal-like PDX model. Flow cytometry histograms show the distribution of the markers indicated in the figure. Filled blue histogram represents EpCAM positive tumor cell population, and the EpCAM negative population (mouse stroma cells) is indicated by the red line. Grey contour represent unstained control.

After enzymatic degradation of PDX tissue the single cell suspensions contained a mix of human and mouse cells. The initial challenge was to separate the human cancer cells from the mouse stroma cells by flow cytometry. When staining with the EpCAM antibody (clone 9C4), the flow analyses showed a defined subpopulation in both xenografts (Figure 1A and B). It was, however, unclear whether all the human cells (i.e. all cancer cells) in the cell suspension were detected using EpCAM, and furthermore, whether the antibody also recognized the mouse version of the antigen. The specificity and sensitivity of EpCAM as a marker of human tumor cells, was tested by triple and quadruple staining combining anti-EpCAM, anti-Tra-1-85 (pan anti-human antibody, filled blue), anti-H2Kd (mouse MHC class I antibody, red line) and Hoecst-3342 (DNA-content, grey contour), with subsequent flow cytometry analysis (Figure 1A and B, right panel). Our results showed that the anti-EpCAM antibody was human specific, and all the human cells in both xenografts were recognized as EpCAM positive. Staining of the luminal xenograft suspension with the anti-mouse MHC class I antibody and the pan anti-human antibody was mutually excluding (Figure 1B right panel). The H2-kd antibody failed to show any binding in the cell suspension from the basal-like tumor, while both Hoechst staining and Tra-1-85 positivity confirmed the human origin of the EpCAM positive cells in both xenografts (Figure 1A).

### EpCAM positive tumor cells from the luminal-like xenograft were tumorigenic

The two PDX models were routinely serially passed as 1-2 mm^3^ pieces implanted orthotopically. The small tumor tissue pieces contain human tumor initiating cell (TIC) population(s), as well as mouse stromal cells. For isolation and functional characterization of TIC subpopulations, it was necessary to test whether the tumor cells maintained their tumorigenic capacity when injected as sorted single cell suspensions without the support of stromal cells. It was also of value to confirm that the EpCAM negative population did not contain TICs. Both EpCAM positive and EpCAM negative cells were therefore isolated using FACS, and re-injected (10^5^ and 10^4^ cells, *in vivo* tumorigenicity assay) in the mammary fat pad of NSG mice. The EpCAM positive cells from the luminal xenograft demonstrated the highest tumorigenic capacity, indicating presence of TICs (Table 1, and growth curves in Figure S1). As expected, the EpCAM negative fraction (containing only mouse stroma cells) did not initiate tumor growth. Stained cells run through the FACS machine but not sorted (all events) were used as control. Interestingly, unsorted basal-like cells gave rise to only one tumor from four separate injections in mice suggesting that these tumor cells might be dependent on support from the stroma cells to initiate tumor growth.

**Table 1:** Test of Tumorigenicity of Cell Fractions from two Breast Cancer Xenograft Models.

Surface marker	EpCAM positive	EpCAM positive	EpCAM negative	EpCAM negative	All events	All events	1∶1 mix EpCAMpos EpCAMneg
No of cells injected	10^5^	10^4^	10^5^	10^4^	10^5^	10^4^	10^5^
**Basal-like**	0/7	0/6	0/9	0/6	1/4	0/6	0/2
**Luminal-like**	7/9	0/3	0/8	0/3	3/7	0/3	3/6

Injection in MFP of NSG mice.

Having established EpCAM as a marker specifying human tumor cells from these two PDX models, we hypothesized that this population was heterogeneous and that it would be possible to identify cell surface markers for prospective detection and isolation of tumor cells with different tumorigenic capacity. To this end, dissociated single cells from both xenografts were stained with EpCAM in combination with a number of markers (Table 2) and IHC analysis of sectioned xenograft tumors was performed (Figure S2). Of all the tested markers, CD24, SSEA-4, SSEA-1 (CD15) CXCR4, E-cadherin and CD44v6 were expressed on the EpCAM positive population of the tumorigenic luminal-like xenograft cells, and thus candidates for defining functionally different luminal tumor cell subpopulations (Table 2). To test whether the candidate antibodies were specifically anti–human, dissociated cells from the luminal-like xenograft were double-stained using anti-EpCAM or anti-TRA-1-85 in combination with either of the markers of interest (Figure 1C). This experiment revealed that expression of some of the markers did not define subpopulations, as all human cells were recognized (CD49b, CD31, CD166). CD44v6 expression was excluded due to variable expression caused by the enzymatic digestion of the tumors [20]. SSEA-1 was excluded due to expression also in the basal-like model (Table 2) in which dissociated cells in suspension showed very low tumorigenicity. We therefore hypothesized that SSEA-1 might not define a population with tumorigenic potential, and chose to proceed with SSEA-4 and CD24 to define subpopulations within the EpCAM-positive fraction of luminal-like xenograft cells.

**Table 2:** Expression of Cell Surface Markers and Aldefluoractivity in EpCAM Positive Cells from Two Breast Cancer Xenografts Models, measured by flow cytometry.

	Luminal-like	Basal-like
Marker	Expression on EpCAM + cells	Expression on EpCAM + cells
**SSEA-4/stage specific antigen 4**	++	0
**CD24/heat stable antigen 24**	++	+++
**CD184/CXCR-4**	++	++
**CD31/PeCAM-1**	++++	0
**CD166/ALCAM**	++++	+
**CD44v6, splice variant of CD44**	+	+
**E-cadherin, cadherin-1,**	++	+
**CD44/receptor for hyaluronic acid**	0	0*
**P75, LNGFR, CD271,**	0	+
**ALDH, aldefluor activity**	+++	++
**CD49f/integrin alpha 6**	0	+++
**Annexin V**	0	0
**CD90/Thy-1**	0	0
**CD34/transmembrane sialomucin family**	0	0
**CD142/tissue factor**	0	++
**NG2, CSPG4, HMW-MAA**	0	+
**CD49b/integrin alpha 2**	++++	0
**CD15, SSEA-1, Lewis 1 antigen**	++	++
**CD45/leucocyte common antigen**	0	0
**CD117/c-kit/**	0	++
**CD133**	0	+++

++++  =  all EpCAM positive cells positive, +++ =  40-90% were positive, ++ =  5–39% were positive, +  =  less than 5% positive cells, 0  =  no cells expressed marker. *  =  was highly positive when xenografts tumor was digested with trypsin.

### SSEA-4 and CD24 are expressed in patient biopsies and define tumorigenic subpopulations in the luminal-like PDX

**Figure 2:**
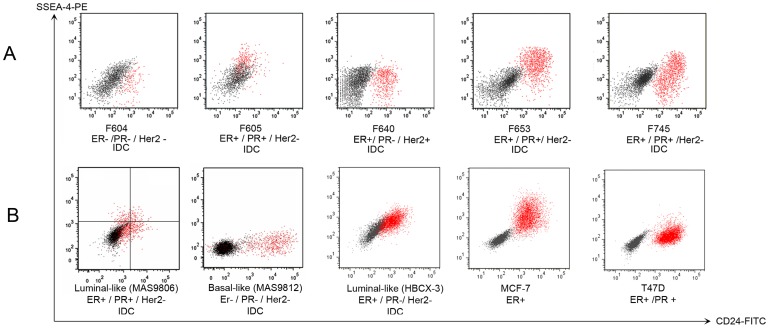
Flow cytometry analysis of EpCAM positive cells from human primary breast tumors, xenografts, and breast cancer cell lines. Freshly harvested primary or xenograft tumor material was minced and digested and the whole cell suspension was stained with anti-EpCAM antibody combined with anti-CD24 and anti-SSEA-4. A) Flow analysis of EpCAM positive cells from five randomly chosen primary breast cancer tumors. F indicate tumor number, ER  = estrogen-, PR =  progesterone-, and Her2- receptor status are indicated under the corresponding dot plot. IDC indicates that primary tumor was invasive ductal carcinoma. B) Flow analysis of EpCAM positive cells from three PDX models and two breast cancer cell lines. The dot plots illustrate the distribution of CD24 and SSEA-4 expressing cells. Red dots are antibody stained cells; black dots represent unstained control.

Expression of CD24 has been associated with breast cancer cells of low tumorigenic capacity [10], and several studies demonstrate that expression of CD24 can classify functionally distinct subpopulations of tumor cells. SSEA-4 is a much less used marker in breast cancer research, but was previously identified as a marker of the ductal zones containing normal stem cells of the breast [21],[22]. It was therefore interesting to investigate whether these markers were co-expressed also in tumors derived directly from patients. Fresh tumor material from five randomly chosen breast cancer patients was disaggregated and triple stained with anti-EpCAM, anti-CD24 and anti-SSEA-4 antibodies. The results (Figure 2A) confirmed that all three markers were, to a varying degree, expressed on primary breast cancer cells, thus, indicating inter-patient variations in cell surface marker expression, even within the EpCAM positive population. Similar analysis of another PDX model (Luminal-like, HBCX-3 [5]), as well as MCF-7 and T47D cells (Figure 2B) confirmed CD24 and SSEA-4 expression in other ER+ breast cancer PDX models and cell lines.

### SSEA-4 and CD24 define tumor cell subpopulations with different tumorigenic ability

**Figure 3:**
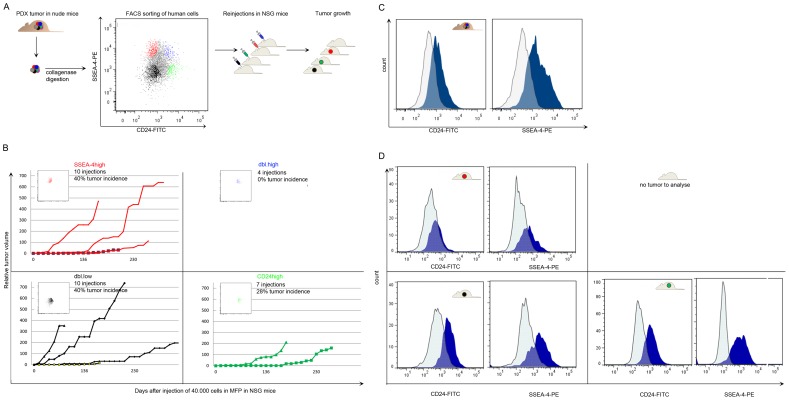
*In vivo* functional characterizations of four EpCAM positive tumor cell subpopulations, defined by CD24 and SSEA-4, from the luminal-like PDX. A) Concept figure illustrating the workflow of the *in vivo* tumorigenicity assays. The FAC-sorted populations are indicated by color in the dot plot. Red indicates SSEA-4^hi^, blue indicate dbl.high, green indicate CD24^hi^ and black dots indicate dbl.low cells. B) Growth curves of tumors resulting from injection of FAC-sorted pure populations. 4×10^4^ cells from each fraction were injected in the right mammary fat pad of NSG γ null mice. Tumor diameter was measured twice each week. C) Flow cytometry analysis of EpCAM positive cells from the “original” tumor. This is the same tumor as in A, but the fluorochrome intensity is here illustrated by histograms, and unstained control cells are included. Harvested tumors were disaggregated and analyzed by flow cytometry after staining with anti- EpCAM, CD24 and SSEA-4 –antibodies. Dark blue histograms indicate the stained samples; light blue contours indicate the unstained control cells. D) Flow cytometry analysis of EpCAM positive cells from tumors in B. Representative histograms are shown.

As Figure 2B and 3A demonstrate, SSEA-4 and CD24 can be used to define four subpopulations in luminal like EpCAM positive cells; SSEA-4^low^/CD24^low^ (dbl.low), SSEA-4^high^ cells (SSEA-4^high^), SSEA-4^high^/CD24^high^ (dbl.high) and CD24^high^ cells (CD24^high^). To test whether they had different capacity for *in vivo* tumorigenicity, FACS was used to isolate pure fractions from each subpopulation, and 40.000 cells from each subpopulation were injected in MFP of NSG mice. Under these conditions, the dbl.high cells did not produce tumors in any of the four experiments while the three other populations did (Figure 3B). The dbl.high subpopulation thus seemed to contain a significantly lower number of TICs than the other fractions. The luminal PDX model is dependent of estrogen for growth, hence, tumor growth cease if estrogen is removed [8]. Immunohistochemical staining for estrogen receptor (ER) positivity showed that both the primary tumor and the PDX contain ER positive and negative tumor cells (Figure S3 upper panel). It was therefore of interest to test whether the dbl.high fraction was depleted of ER positive cells, and thus unable to respond to the growth stimulatory signals from estrogen. ER staining of sorted subpopulations showed that they all contained ER positive cells, demonstrating that absence of ER in the dbl.high subpopulation could not explain their low tumorigenic capacity (Figure S3).

### SSEA-4^high^ cells were tumorigenic, but did not give rise to CD24^high^ cells

The cancer stem cell hypothesis proposed that cancer stem cells should have the ability to reproduce the original tumor mass. To elucidate the self renewal capacity of the subpopulations, three tumors from the SSEA-4^high^ and dbl.low populations, and two tumors from the CD24^high^ populations (generation P0) were disaggregated and analyzed for expression of EpCAM, CD24 and SSEA-4 (Figure 3D). Tumors initiated from the dbl.low and CD24^high^ populations were similar to the original xenografts, expressing all three markers (Figure 3D bottom panels compared to 3C). However, the tumors initiated from SSEA-4^high^ cells (red dots in Figure 3A and red lines in Figure 3B) did not contain CD24^high^ cells; hence the SSEA-4^high^ population seemed to harbor TICs that could not recapitulate the original tumor (Figure 3D upper panel compared to 3C). Tissue from tumors originating from all three subpopulations was, furthermore, implanted in the MFP of NSG mice and they were all able to establish and grow (generation P1, results not shown).

### Whole Genome Expression profiling revealed a gene set predicting longer overall survival in clinical samples

**Figure 4:**
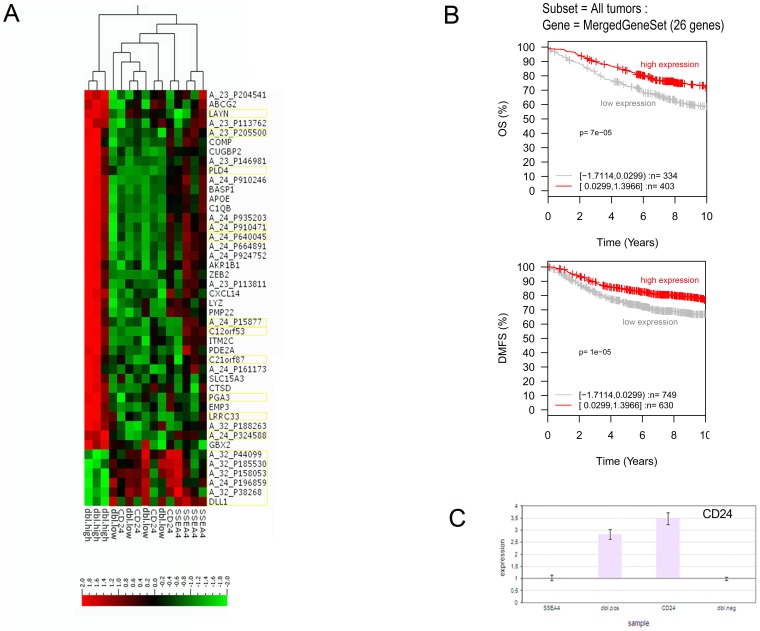
Whole genome expression analyses of sorted tumor cell subpopulations. EpCAM positive cells from the luminal xenografts were separated based on expression of SSEA-4 and CD24 using FACS. A) Normalized gene expression data from all 15 samples were subjected to t-test comparison of two groups (dbl.high subopoulations vs. the tumorigenic subpopulations) with p≤0.004 and FDR = 0.2. The figure shows a cluster heatmap of the 44 significantly differentially expressed genes. Probes in yellow frames are not included in B, either because they are not annotated, the genes could not be found in GOBO, or they showed lower expression in the dbl.high population. The A_32_P188263 probe maps to the C1QB gene, which is already represented in the 26 gene list. B) Kaplan-Meier analysis using overall survival (OS) and distant metastasis free survival (DMFS) as endpoint and 10-year censoring as displayed in GOBO. C) Total RNA was isolated from FAC-sorted subpopulations and RT-qPCR was performed using primers against CD24. The bars illustrate the fold difference.

The four subpopulations showed phenotypical and functional differences, and we next aimed to relate the tumorigenic potential to differences in transcriptional profiles. Total RNA was isolated from the four FAC-sorted subpopulations (dbl.low, SSEA-4^high^, dbl.high and CD24^high^), and analyzed using whole genome expression arrays. Unsupervised clustering of the 1000 most variable genes revealed high similarity between the four subpopulations. Considering that these comparisons were performed between cells originating from the same tumor and with relatively homogenous EpCAM expression (Figure 1), and with no previous selection from *in vitro* cultivation, this might be expected. The main variance across the populations was caused by genes highly expressed in the dbl.high population. This corresponded well to the functional data. A two group comparison using t-test (Figure 4A) revealed 44 differentially expressed transcripts (p≤0.004, FDR = 0.20), of which 6 genes were less expressed in the dbl.high compared to the other populations (Figure 4A and Table 3). The online database “Gene expression-Based Outcome for Breast Cancer” (GOBO) [19] was used to test the association between the set of genes highly expressed in the non-tumorigenic population with outcome for 1881 breast cancer patients. 26 genes from the 44-list were both highly expressed in dbl.high cells and represented in the GOBO database. In tumors, high expression of the 26 genes, correlated significantly with longer overall survival (OS) (N = 737, p<0.0001) and distant metastasis free survival (DMFS) (N = 1379, p<0.0001) in Kaplan-Meier analysis (Figure 4B). The 26 gene set, furthermore, correlated with longer DMFS in multiple subgroups of the 1881 sample breast cancer dataset; in basal-like, ER-negative, LN-negative, as well as in grade 2 tumors, high expression of the 26 genes predicted lower risk of recurrent disease and longer DMFS. RT-qPCR validation confirmed the stringency of the FACS procedure and microarray gene expression analysis. This was exemplified by the CD24 gene which was found highly expressed in the CD24 expressing populations, both by microarray analysis and by RT-qPCR (Figure 4C).

**Table 3:** List of the 44 probes and corresponding genes significantly differentially expressed in the non-tumorigenic population compared to the tumorigenic populations.

ProbeID, highly expressed, used in GOBO	Gene Symbol	Gene name
*A_23_P18713*	*ABCG2*	ATP-binding cassette, sub-family G (WHITE), member 2
*A_23_P258190*	*AKR1B1*	aldo-keto reductase family 1, member B1 (aldose reductase)
*A_23_P164650*	*APOE*	hypothetical LOC100129500; apolipoprotein E
* *A_23_P113762*	*S100B*	S100 calcium binding protein B
* *A_23_P113811*	*RPL19*	ribosomal protein L19; ribosomal protein L19 pseudogene 12
* *A_23_P146981*	*CTSZ*	cathepsin Z
* *A_23_P204541*	*CNTN1*	contactin 1
* *A_24_P161173*	*PDE4D*	phosphodiesterase 4D, cAMP-specific (phosphodiesterase E3 dunce homolog, Drosophila)
* *A_24_P664891*	*COX6A1*	cytochrome c oxidase subunit VIa polypeptide 1
* *A_24_P910246*	*TCN2*	transcobalamin II; macrocytic anemia
* *A_24_P924752*	*LGALS2*	lectin, galactoside-binding, soluble, 2
* *A_24_P935203*	*UBC*	ubiquitin C
*A_23_P213385*	*BASP1*	brain abundant, membrane attached signal protein 1
*A_23_P137366*	*C1QB*	complement component 1, q subcomponent, B chain
*A_24_P264943*	*COMP*	cartilage oligomeric matrix protein
*A_23_P52556*	*CTSD*	cathepsin D
*A_23_P202071*	*CUGBP2*	CUGBP, Elav-Like Family Member, CELF2
*A_23_P213745*	*CXCL14*	chemokine (C-X-C motif) ligand 14
*A_23_P119362*	*EMP3*	epithelial membrane protein 3
*A_23_P131183*	*GBX2*	gastrulation brain homeobox 2
*A_24_P402690*	*ITM2C*	integral membrane protein 2C
*A_24_P42264*	*LYZ*	lysozyme (renal amyloidosis)
*A_23_P401106*	*PDE2A*	phosphodiesterase 2A, cGMP-stimulated
*A_23_P100711*	*PMP22*	peripheral myelin protein 22
*A_23_P75786*	*SLC15A3*	solute carrier family 15, member 3
*A_24_P367454*	*ZEB2*	zinc finger E-box binding homeobox 2
**Probe ID, less expressed in dbl.high compared to the tumorigenic populations**		
*A_32_P44099	GUCY1A2	guanylate cyclase 1, soluble, alpha 2
*A_32_P185530	RAPGEF5	Rap guanine nucleotide exchange factor (GEF) 5
*A_32_P158053	UBA6	ubiquitin-like modifier activating enzyme 6
*A_24_P196859	STS	steroid sulfatase (microsomal), isozyme S
*A_32_P38268	BAT2L	PRRC2B (proline-rich coiled-coil 2B)
A_23_P167920	DLL1	delta-like 1 (Drosophila)
**Probe ID, not found in GOBO**		
*A_23_P424603*	*C12orf53*	*chromosome 12 open reading frame 53*
*A_23_P372368*	*C21orf87*	*chromosome 21 open reading frame 87*
*A_23_P127565*	*LAYN*	*layilin*
*A_23_P88222*	*PLD4*	*phospholipase D family, member 4*
* *A_32_P188263*	*C1QB*	*complement component 1, q subcomponent, B chain*
*A_23_P132856*	*LRRC33*	*leucine rich repeat containing 33*
*A_23_P150547*	*PGA3*	*similar to Pepsin A precursor; pepsinogen 3, group I (pepsinogen A); pepsinogen 4, group I (pepsinogen A)*
* *A_24_P910471*	*ACAA1A*	*acetyl-CoA acyltransferase 1*
**Probe ID, no annotation found**		
*A_23_P205500*		
*A_24_P15877*		
A_24_P324588		
*A_24_P640045*		

The 26 top genes were subjected to gene set analysis on breast cancer patient outcome in the GOBO database [19].

Probes downregulated in dbl.high.

*Probes upregulated in dbl.high. The top 26 gene IDs were put in gene set analysis on tumors in the GOBO breast cancer gene expression database.*

*Genes annotated by BLAST of probe sequence.

### Genomic aberrations could not explain the non-tumorigenic phenotype

**Figure 5:**
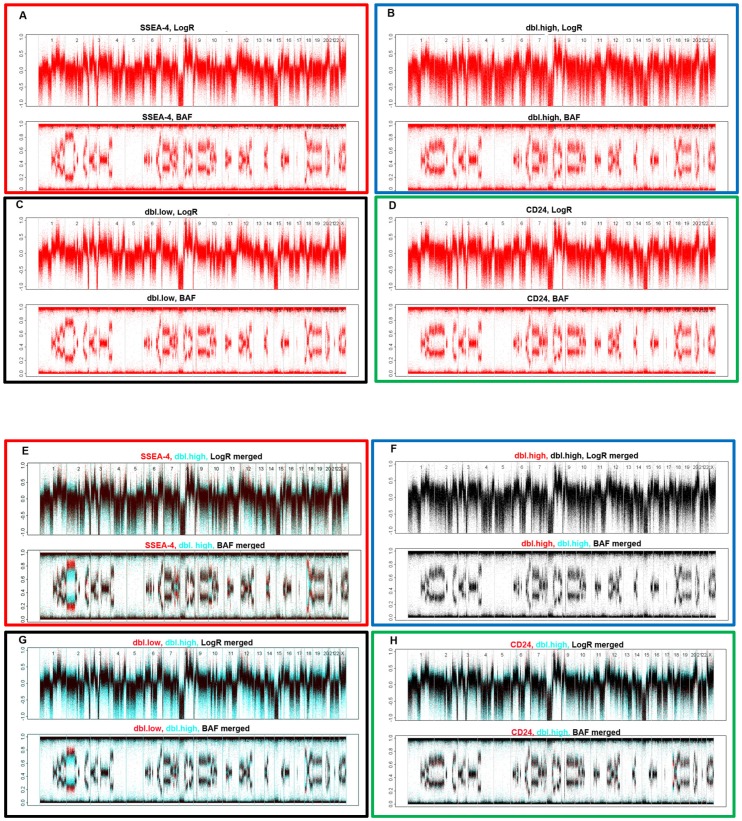
SNP array data displayed as unsegmented dotplots. Total signal intensity (LogR) and the B allele frequency (BAF) from all four subpopulations are shown (A–D). For illustration of similarities and differences in genomic aberrations, overlay images comparing LogR and BAF from each population to the dbl.high population are shown (E–H). Light blue color indicates copy number pattern observed only in dbl.high population, red color indicates pattern observed only in the cell populations to which dbl.high is compared, and black indicates identical LogR and BAF.

The functional differences between the four subpopulations made it interesting to screen for associated genomic aberrations utilizing SNP arrays. All four subpopulations displayed similar copy number aberrations characteristics for the luminal B breast cancer subtype. These genetic alterations included focal amplification of 8p11-12, 11q13 (*CCND1*), 12q15 (*MDM2*) and multiple focal amplicons distal to *ERBB2* on chromosome 17q (Figure 5). Cell surface expression of CD24 seemed to divide the EpCAM positive cells in two slightly different variants with respect to copy number variations. The B allelic frequency (BAF) plot of chromosome 2 was similar in the CD24 expressing populations and differed from CD24 negative populations (Figure 5 A and C compared to B and D). Furthermore, on chromosome 18, distinct differences in BAF were evident (Figure 5E-H). Importantly, none of the observed differences in genomic aberrations were unique for the non-tumorigenic population (Figure 5E, G and H). This indicates that although the cell surface marker based FAC-sorting enriched for functional differences between the populations, underlying genomic differences were not determinants of the observed *in vivo* tumorigenicity.

## Discussion

Intratumoral heterogeneity may explain why systemic cancer therapies fail, and many attempts have been made to study, especially genomic, heterogeneity in patient samples [23],[24]. Studies of non-genetic molecular variations and correlations with tumorigenic abilities (functional heterogeneity) are, due to limited supply of material, literally impossible to perform in clinical samples. The intratumor heterogeneity of a representative PDX model was therefore explored. Clinically relevant cell surface markers were used to prospectively define four subpopulations, and *in vivo* functional heterogeneity was measured. Finally, transcriptional and genomic characteristics were analysed with emphasis on differences between the populations that could potentially explain their divergent *in vivo* tumorigenicity.

The *in vivo* assays were performed as four separate biological parallels, and the purity and viability of the fractions were controlled before each injection. Even though the common genetic origin of the four populations was confirmed by the copy number analyses, no tumors were formed after any of the injections of the dbl.high (CD24^high^/SSEA4^high^) fractions suggesting that this cell population had a lower intrinsic tumorigenic potential than the others. Interestingly, tumors resulting from injections of pure dbl.low - and pure CD24^high^ cells were, when re-analyzed by flow, always similar to the original PDX tumor. In other words, cells expressing neither of the cell surface markers, or only CD24, had the ability to form offspring containing all four subpopulations, and did so every time. Conversely, cells expressing only the SSEA-4 marker, although tumorigenic, were never able to regenerate all four subpopulations in the resulting tumors. Altogether, these results indicate that the TICs in the SSEA-4^high^ population were different from the TICs in dbl.low and CD24^high^ populations. We therefore hypothesize that tumor initiating cells from this PDX had more than one phenotype, and the TICs within each subpopulation did not vary from experiment to experiment. In line with this, it has been shown that from the same cell line, both phenotypically pure luminal-like cells as well as their more stem cell-like counterparts could give rise to tumors [25],[26]. This PDX model, always regenerates the four defined subpopulations of the original PDX when routinely implanted as small tumor tissue pieces in MFP, and the morphological characteristics, receptor status and transcriptional profile are stable over multiple passages [7]. Therefore, the SSEA-4^high^ population does probably not dominate over the dbl.low or CD24^high^ population; otherwise, the CD24 expression would, over time, be lost. Considering that SSEA-4 is regarded as a stem cell marker, this result was unexpected. One possible explanation might be that CD24 expression was redundant for developing new tumors from the SSEA-4^high^ cells, or that the daughter cells had lost their ability to differentiate along the luminal-epithelial lineage, and hence did not express CD24. It is tempting to speculate that cells expressing SSEA-4 could be locked into myoepithelial differentiation.

From the tumorigenicity assays and re-analysis of the resulting tumors, it was clear that the four subpopulations represented three different functional phenotypes; the non-tumorigeic, the PDX recapitulating, and the tumorigenic but non-CD24 recapitulating. In search for transcriptional patterns explaining the functional phenotypes, whole genome expression analysis was performed. Corresponding well with the *in vivo* data, the dbl.high cell population was the population that seemed to be most different from the others. Collectively, the genes differentially expressed in this population compared to the other cell populations, do not point to a specific tumor inhibiting function or cellular target. It is therefore interesting that when using the genes highly expressed in the non-tumorigenic population as a marker for patient stratification in the online GOBO database, patients with high expression of this gene set experienced improved overall survival and distant metastasis free survival in a multivariate analysis. This finding is in concordance with our *in vivo* functional data, and suggests that the genes highly expressed in the non-tumorigenic cells may affect tumorigenicity when highly expressed in clinical samples. Although, high expression of the 26 genes set in primary breast cancer cells seemed to indicate less aggressive cancer, it does not necessarily indicate expression of the two cell surface markers.

SNP arrays were employed to delineate whether copy number alterations were specifically assigned to each population. Interestingly, genomic heterogeneity within each population was revealed, indicating that the CD24 and SSEA-4 based FAC-sorting did enrich for functional phenotypes, but perhaps not for underlying genetic aberrations. It is possible that transcriptional differences between the TICs within each subpopulation might be camouflaged by transcription from intermixed less tumorigenic cells. It is also possible that the tumor initiating capacity might be better explained by differences in the post-transcriptional or protein regulation mechanisms. The clinical impact of the genes highly expressed in the dbl.high non-tumorigenic population may indicate that the observed phenotypic and functional heterogeneity within this tumor most likely does not result from genetic changes. Recently, Snuderl and colleagues [24] explored the genetic intratumoral heterogeneity of glioblastomas. In a comment to their work [27], different scenarios for interactions between cellular subclones within tumors were discussed. Data presented here suggest that in this particular model, a so-called “random” scenario, where subclones of cancer cells survive autonomously, is the most probable. While one population could not give rise to new tumors, three populations were tumorigenic, independently of each other, and independently of stroma cells. Intriguingly, the three tumorigenic subpopulations, also gave rise to two phenotypically different types of daughter tumors, implying possible functional variations between the resulting tumors. This study emphasizes the need for broad attacks against multiple subpopulations within the primary tumor to obtain systemic eradication of the disease.

## Supporting Information

Figure S1 Growth curves of tumors resulting from injection of 10^5^ FAC-sorted cells from the luminal-like PDX in the MFP of NSG mice. Upper chart: EpCAM positive cells; out of nine injections, tumor was formed in seven. Middle chart: 2.5×10^5^ EpCAM positive cells were mixed with 2.5×10^5^ EpCAM negative cells and injected. Of six injections, tumors formed in three. Lower chart: 10^5^ cells run through the FACS Diva, but not sorted, were injected. Of seven injections, three tumors were formed.
(TIF)

Figure S2 Expression of relevant markers in basal-like and luminal-like orthotopically growing breast cancer xenografts models. A) Immunohistochemistry (IHC) on sections from paraffin embedded tumors from the basal-like (upper row) and luminal-like (lower row) PDX. The sections were stained with antibodies to the proteins indicated. The antibodies shown did not react with mouse stromal cells. B) Immunofluorecent staining of frozen tissue sections from the two PDX models as indicated.
(TIF)

Figure S3 Bright field images of immunohistochemical staining for estrogen receptor in paraffin embedded sections from the original primary tumor (upper left side), the luminal-like PDX model (upper right side), and stained cell suspensions from each of the four subpopulations (Lower panel). The FAC-sorted pure cell suspensions were placed on glass slides, fixed and stained. Cells showing positive staining for ER are brown; the cell nuclei were counterstained with hematoxylin (blue). Arrows point to ER positive cells.
(TIF)
