# Supplementary figures and images for: Differential In Vivo Tumorigenicity of Distinct Subpopulations from a Luminal-Like Breast Cancer Xenograft
Source: PLoS One. 2014 Nov 24;9(11):e113278. doi: 10.1371/journal.pone.0113278 (PMC4242648; doi:10.1371/journal.pone.0113278)

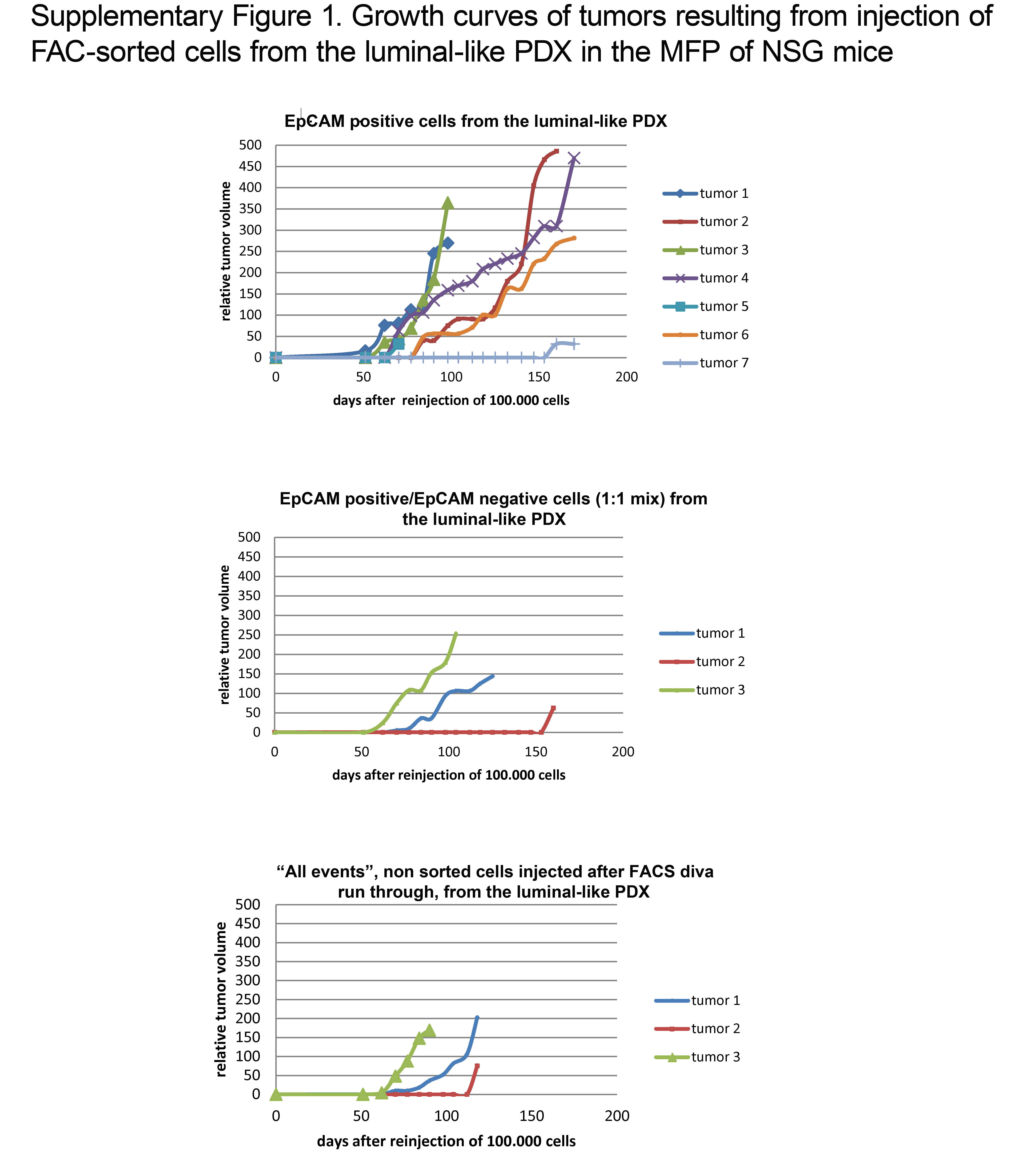

Supplement: Figure S1 — Growth curves of tumors resulting from injection of 105 FAC-sorted cells from the luminal-like PDX in the MFP of NSG mice. Upper chart: EpCAM positive cells; out of nine injections, tumor was formed in seven. Middle chart: 2.5×10^5^ EpCAM positive cells were mixed with 2.5×10^5^ EpCAM negative cells and injected. Of six injections, tumors formed in three. Lower chart: 105 cells run through the FACS Diva, but not sorted, were injected. Of seven injections, three tumors were formed. (TIF) [file pone.0113278.s001.tif]

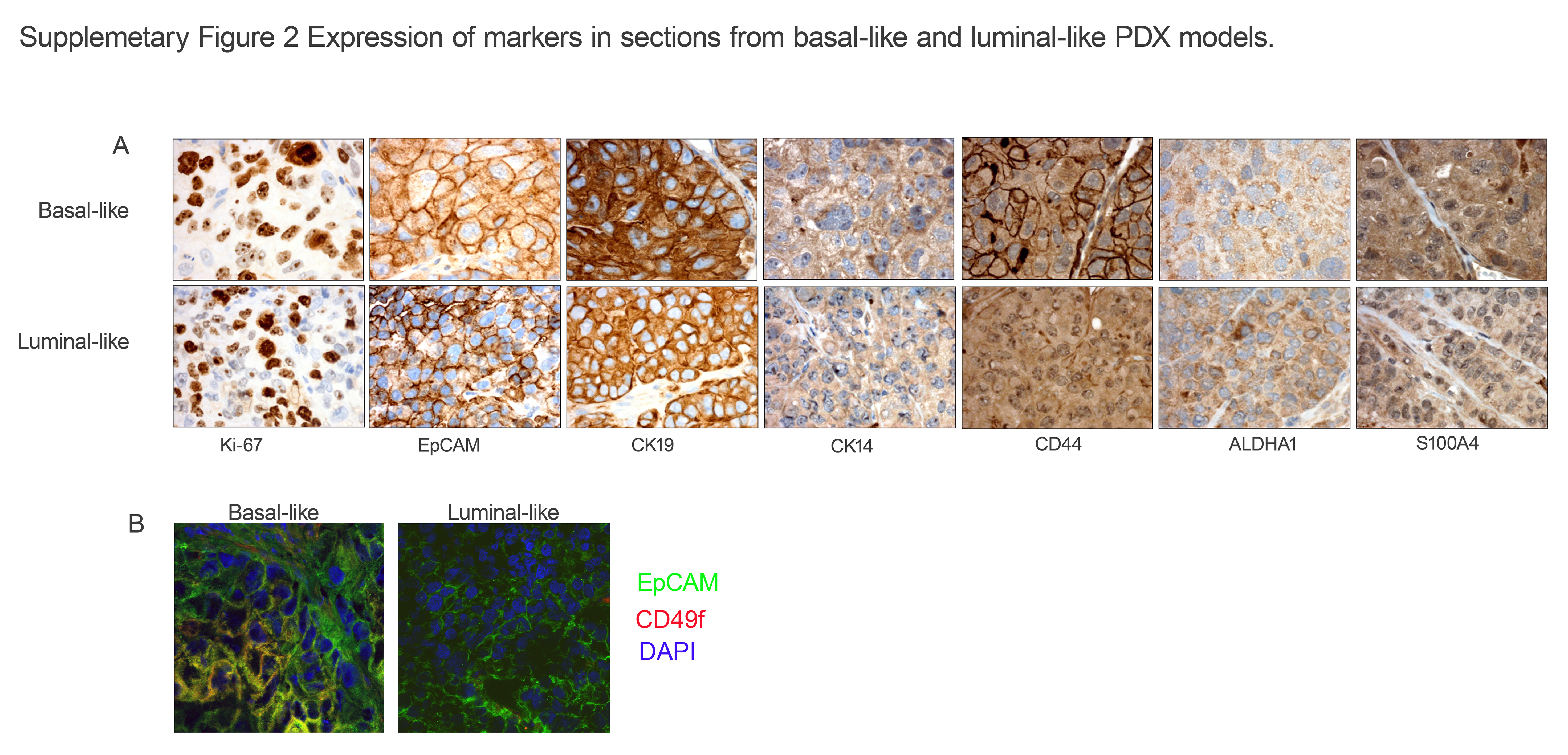

Supplement: Figure S2 — Expression of relevant markers in basal-like and luminal-like orthotopically growing breast cancer xenografts models. A) Immunohistochemistry (IHC) on sections from paraffin embedded tumors from the basal-like (upper row) and luminal-like (lower row) PDX. The sections were stained with antibodies to the proteins indicated. The antibodies shown did not react with mouse stromal cells. B) Immunofluorecent staining of frozen tissue sections from the two PDX models as indicated. (TIF) [file pone.0113278.s002.tif]

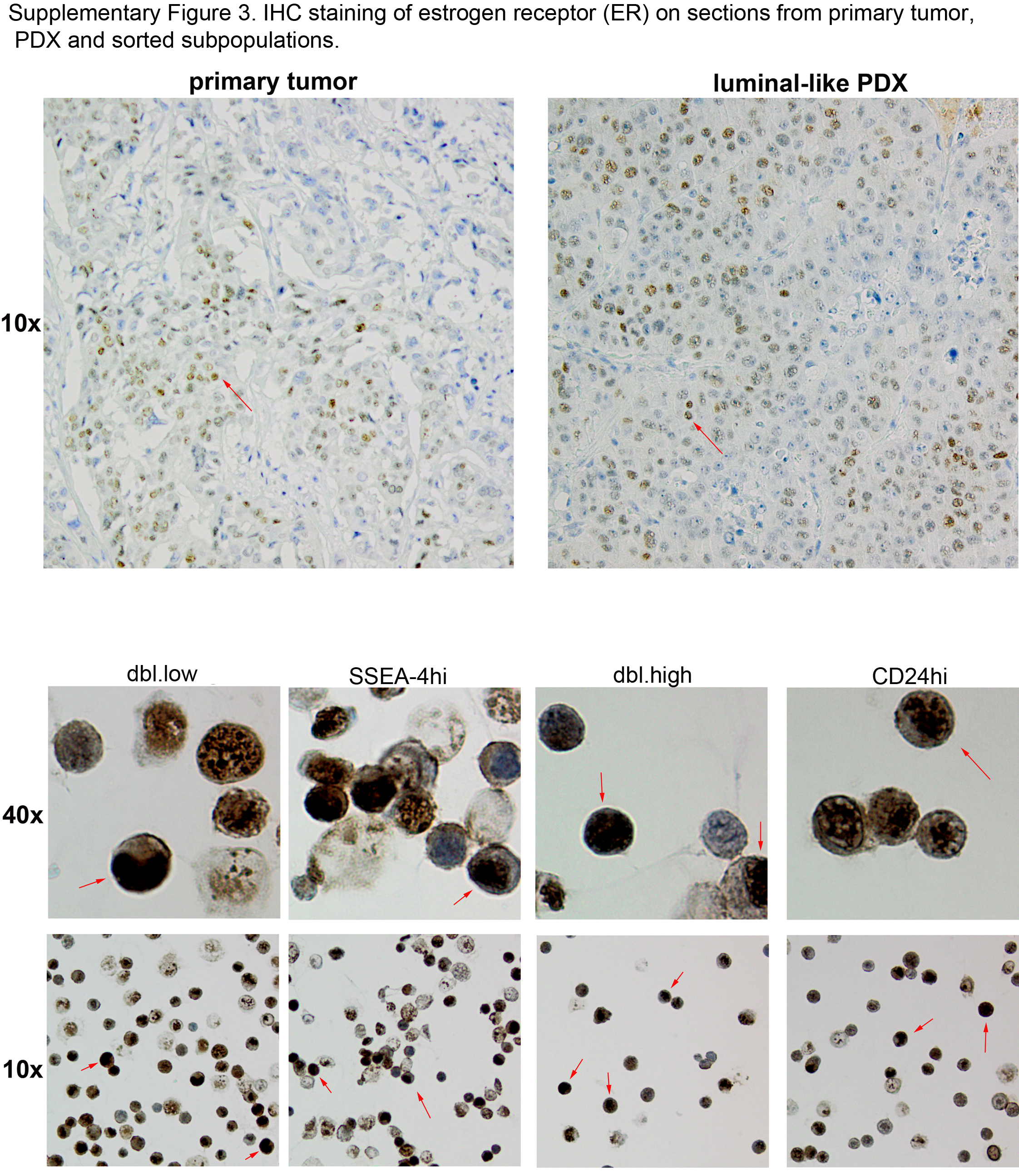

Supplement: Figure S3 — Bright field images of immunohistochemical staining for estrogen receptor in paraffin embedded sections from the original primary tumor (upper left side), the luminal-like PDX model (upper right side), and stained cell suspensions from each of the four subpopulations (Lower panel). The FAC-sorted pure cell suspensions were placed on glass slides, fixed and stained. Cells showing positive staining for ER are brown; the cell nuclei were counterstained with hematoxylin (blue). Arrows point to ER positive cells. (TIF) [file pone.0113278.s003.tif]
